# Using a custom mobile application for change management in an electronic health record implementation

**DOI:** 10.1093/jamiaopen/ooz048

**Published:** 2019-12-16

**Authors:** Tony Threatt, Claude J Pirtle, Jennifer Dzwonkowski, Kevin B Johnson

**Affiliations:** 1 HealthIT, Vanderbilt University Medical Center, Nashville, Tennessee, USA; 2 Department of Biomedical Informatics, Vanderbilt University Medical Center, Nashville, Tennessee, USA

**Keywords:** electronic health record, change management, EHR implementation, mobile application, implementation science

## Abstract

**Objectives:**

Institutions cite managing the modification in infrastructure, technical support, and process change as substantial barriers to a successful electronic health record (EHR) implementation. In an effort to organize and centralize the complex scheduling, task completion and communication needs of a “big-bang” EHR go-live, we developed a unified communication system with the goal of improving implementation process efficiency. Our goal was to create a platform that would work across the medical enterprise.

**Materials and Methods:**

We employed an agile process to design the application, called Hubbl, based on initial requirements and iteratively obtained stakeholder user. The final feature set included role-specific organization, integrated communication, task and content management tooling, and embedded project information retrieval, all embedded into the end user’s day to day activities.

**Results:**

User enrollment continually increased from launch in February of 2017 through go-live day. During the pre-go-live period, usage increased from an average of 7.37 events/user/day to 18.65 events/user/day with over 97 communications sent across all periods. 5400 unique users accessed tip sheets and information retrieval tools averaged 28.84 searches/user/day during the go-live period with an average high of 46.33 searches/user/day 5 days post-go-live. User access during go-live and post-go-live averaged 12.82 accesses/user/day and decreased from 20.42 average accesses on day one of go live to 14.07 averaged accesses on day 60 of post-go-live with over 727 tasks monitored to completion during all periods.

**Conclusion:**

Hubbl was an essential component of our communication, task coordination, and change management strategy, for our EHR go live. Institutions that choose a unified mobile and web-based platform during a substantial IT (information technology) implementation can feasibly ensure task completion, project coordination, and timely information dissemination.

## INTRODUCTION

*It is not the strongest or most intelligent who will survive, but those who can best manage change – Charles Darwin*

In the case of information technology adoption and diffusion, the major challenges to successful change as noted by Lorenzi, “are often more behavioral than technical.”^1^ An organization’s inability to manage enterprise-wide change may lead to adverse health events, especially with health record change.^2^ Institutions cite managing the modification in infrastructure, technical support and inevitable process changes as major barriers to a successful electronic health record (EHR) implementation,^3^ and patient and clinician dissatisfaction after the implementation.^4–7^

Creating communication goals, priorities and events happening in and around any medical system is necessary to maximize the efficiency and quality of the implementation.^8^ Stakeholders working within this system need to understand what is changing and when, how that change is being managed, and who is responsible for each aspect of change.^9^

Traditionally, organizations communicate through daily emails, printed documents, and town hall meetings.^10^ Although these strategies efficiently deliver “just in case” content, they either become victims of overuse or suffer from being marginalized relative to other staff priorities.^11^^,^^12^

In contrast, the use of mobile phones for just-in-time notification has been well-received as a communication medium. One of the capabilities of mobile phones is to allow a user to receive a notification message in real time.^13^ Multimodal data entry capabilities—using keyboard, touch, or voice are familiar to users. These capabilities improve the process of technology implementation and process change management. For example, real-time end-user feedback could support the identification of problems^14^ that occur on the front line quickly and efficiently. This dynamic feedback empowers the leadership teams to promptly develop a strategy to overcome site-specific issues.

Furthermore, mobile technologies are well designed to allow the creation and management of tasks.^15^ People use mobile devices more than ever to access web content, and the trend continues to increase.^16^ Traditionally, teams tracked tasks through a spreadsheet or a task distribution checklist system. This type of system allows the opportunity to track tasks on a macroscale but can become cumbersome on a more granular level due to an organization’s size. Word processing programs and spreadsheets placed on a shared drive have been used in the past to log and track the training of staff.^17^ These ways do not offer real-time mobile feedback about your previously completed training, the ability to seamlessly schedule training, or a window to view upcoming training courses. Perhaps most importantly, virtually every member of our implementation community owned a smartphone, thereby being equipped to leverage an application designed for this purpose.

As noted above, various disparate tools address task management, secure real-time communication, and knowledge transfer in the field; however, we struggled to identify an integrated suite of tools that satisfied our needs to manage the go-live of a new inpatient, ambulatory, and financial management system across four hospitals and over two hundred clinics spanning a three-state area in the time allotted. Given the unique qualities of mobile technologies including voice, image capture, and keyboard, we believed that a smartphone application designed to manage the implementation would streamline the process by obviating the need for spreadsheets, emails, and shared document drives. The purpose of this article is to describe the design objectives, capabilities, adoption, and provide usage statistics of a mobile application that assisted in our large-scale EHR implementation and change management process.

## METHODS

### Setting

Since 1996, the staff and clinicians at Vanderbilt University Medical Center (VUMC) relied on a locally developed EHR called StarPanel.^18^ This system was firmly ensconced into the culture at VUMC, with high levels of satisfaction over the past two decades.^19^ However, given both the growth of the medical center and the rapidly changing administrative and regulatory requirements of health care, VUMC decided to replace its EHR with a commercially available system, and use this vendor-supplied system for inpatient, outpatient, and revenue cycle management.

### Design objectives

A specific change management challenge drove our design objectives—migrating from a home-grown EHR to a commercial product, and the communication tasks inherent in that migration. We wanted to create a product that allowed communication to occur at the right time, place, and was flexible to our organizational hierarchy to resolve many of the issues of implementation coordination and communication. Our review of commercially available software that we either already licensed (eg, Sharepoint) or could purchase did not provide assurance that one product would meet all of our needs.

### Categorizing users of the app

In a complex healthcare organization, it is common for each individual to be a member of multiple separate teams with differing missions. This application needed to embrace a health system comprised of numerous relationships among people, sites of care, and roles within those sites. Importantly, to ensure that accountable individuals track the receipt of communications or the completion of tasks, the implementation team assigned team leaders these aspects of the project. For example, a nurse may work in a highly specialized medical department (eg, multiple sclerosis clinic) while also reporting to nursing leadership. That same nurse could have a leadership role that reports into clinic management. This approach would support the use of dashboards to ensure that specific groups completed part or all of their assigned work.

### Embedding project communication into routines

Based on previous large-scale change efforts, one of our go-live core principles included removing work management from spreadsheets (which suffer from version control issues) and email (which is difficult to filter based on subcomponents of a major program). We wanted users to be able to receive and complete work, and then broadcast work completion easily. We disseminated tasks to users and published them on a readily available dashboard for medical center leadership to review. We believed that site leaders needed to mark the tasks as completed once a review of the tasks with the end user had occurred to ensure full adoption of this process.

### Communicating completed work throughout the implementation process

Organizations who deploy relevant, timely, easily retrievable, and efficient communication are more likely to succeed in change management. Mobile platforms offer this capability through native functionality such as alerts and persistent (badging) notifications. We believed that to communicate efficiently and effectively, discussions need to occur at the right time and be organized into the daily routine of the individual. We also recognized that the types of work and routines would change as the project progressed. We organized the project into three distinct phases, each of which would have different organizational pressure and routines to consider:

1. Pre-go-live work, such as approving workflows, completing training, and reviewing role-specific information. We realized that the system needed to support both data entry by end users and data retrieval (including the entry of assigned tasks) by leadership.
2. Go-live work, such as managing change requests, receiving feedback from key stakeholders, disseminating training material, supporting system evaluation and communicating workarounds for poorly functioning components
3. Post-go-live work, such as notifying people about education sessions and providing easy access to a help document repository available to all end users as they rotated on and off various services.

### Integrating work functions into one app

In a multihospital system, there are many digital tools available to employees that might be essential to integrate with our application. Some of these tools included our system for recording and managing user-identified problems, knowledge repositories, the training system, and an evaluation tool. Rather than requiring users to download each of these components, we believed it would be optimal to integrate these tools into the application to reduce the workload on the burdened faculty and staff. Ultimately, we were unable to provide the training modules through Hubbl because the vendor system was not mobile ready.

## SYSTEM DESCRIPTION

**Figure 1.**
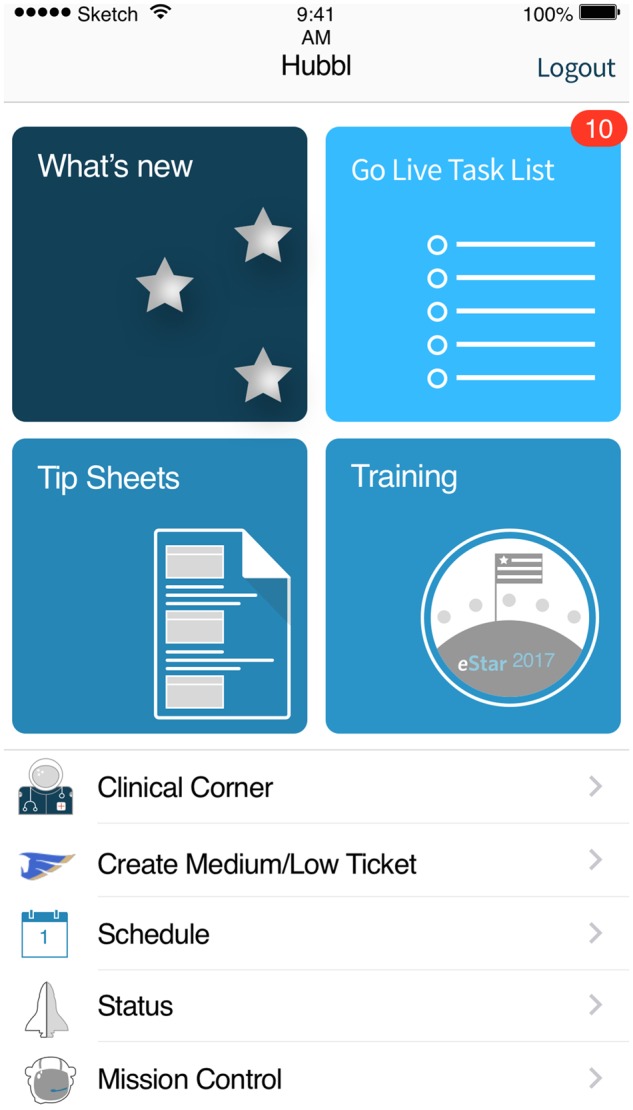
is a screen shot of the splash screen when first entering Hubbl using a mobile device (iOS). The main functionality—what’s new, task list, tip sheets, and training—are at the top of the app and supporting features are in the list below.

Our design result is an application called Hubbl. We chose this name to reflect the similarity of its mission to that of the Hubble telescope, paraphrased as gathering information from the cosmos to better understand the universe around us. In the case of our project, the “cosmos” was the space separating the implementation team from the various clinical and revenue management environments they served. Figure 1 is an example of Hubbl from one mobile platform.

The components of Hubbl include a mobile platform (iOS, Android) and a web site for both administrators and other users. The program provides a user experience conceptually similar to that of most mobile apps, including the use of notification messages, app icon badges to signify new content, and software auto-updates to ensure that the user did not have to keep track of new versions of an evolving app.

Hubbl’s feature set evolved iteratively based on user feedback and requirements, as well as in alignment with the phases of the go-live. The table below summarizes these phases and the description for each feature. We describe some of the most critical functionalities below (Table 1).

### Pre-go-live features

#### Group assignment

We incorporated role management into Hubbl’s administrative functions to manage the group task assignment requirement. Although we initially planned to use titles used by our human resources group to create Hubbl roles, we found that formal titles were either too vague or too confusing to be useful. Instead, we developed an administrator application to assign roles. We combined one group topology, based on physical location, with another topology based on job function/interest. We provided each enrolled Hubbl user with one or more role definitions that facilitated the assignment of tasks by our leadership and mapped formal human resource titles to these roles. For example, peer trainers. This strategy resulted in 546 physical location groupings organized by our three hospitals, 46 patient access and revenue management groups, 44 research groups, and 20 job function groupings.

#### Communication

We designed the communication system to be similar to the mobile email user experience. The user viewed a list of messages and selected any message to expose greater detail, including images and URLs. Users flagged messages as a “favorite” and then could filter unflagged messages from view. Because we did not want users to view this system as a replacement for email, our initial design supported only messages from a central group out to users. We also incorporated a message communication into the administrator interface to easily identify locations/groups/job functions to receive each message.

After approximately 6 months of production use, and thinking through post-go-live needs, our user community requested bidirectional communication within Hubbl to collect go-live status updates from the field based on an implementation leadership query. In response, we added two features to our communication infrastructure: (1) the ability to create ad hoc groups (such as clinicians); and (2) the ability for a member of a group to send a message to all other members of that group from the application.

#### Task list

We based our task list on typical “to do” functionality in most mobile applications. When we created new tasks, we also took advantage of the notification system. A critical functional distinction between Hubbl and typical task management apps was that we integrated this task list with a Tableau Dashboard so that administrative leaders could monitor location, group, or individual progress and be able to assist teams who needed help completing work. Leadership across the medical enterprise reviewed the dashboard during weekly Leadership Accountability Meetings led by VUMC Operational Leadership, and also could access the dashboard from their own accounts.

##### Workflow review tasks

Workflow review tasks ensured that site leaders, across the VUMC system, were reviewing the workflow-related changes that would occur within their specific areas or globally at the go-live. These changes were not explicitly listed in the tasks; rather, the tasks contained a URL pointing to a flow chart that graphically depicted the workflow.

##### Documentation and nondocumentation tasks

Hubbl allowed leadership to target education material, announcements about meetings, and other go-live readiness tasks to groups or all of the medical enterprise. Of note, because this feature linked to specific users, we were able to use dashboards to identify users who had not acknowledged receipt of these tasks.

#### Training information

We integrated Hubbl with our learning management system, absorb learning exchange, through a commercially available application programming interface (API) to show users what training they needed to complete before they were able to access the system after implementation go-live. The user could see what training they needed to finish, the date and time for the training, and the location. The user could also view any digital modules that she needed to complete.

### Go-live features

#### End user change requests

We were able to create an API from our homegrown help desk ticket support system to Hubbl—a feature now available, but seldom used in other ticketing systems. In our case, the API allowed users to complete tickets for incident notification or feature requests, as well as to track ticket status within the application. This feature facilitated constant communication back to the teams who were implementing the EHR within the existing routines of end users.

#### Support evaluation

During the implementation, we had a team who visited sites to evaluate the implementation. The team would fill out an evaluation form at the end of their visit. After filling out this form, it would be published on a Tableau dashboard so that leadership could see which sites had the most substantial opportunity for improvement.

### Post-go-live features

#### Post implementation Frequently Asked Questions (FAQ)

Both during and after implementation, users needed an FAQ capability to understand how to use the EHR to help them through their work. To do this, we provided access for end-users to our content management system. We placed user-friendly views inside the application so that users could find the documentation effectively. We also allowed users to denote “favorite” documents to ease searching for commonly used capabilities or workflows. With this implementation, we realized that we created a large volume of help documents requiring that we develop an organizational reconciliation process.

## EVALUATION

To evaluate Hubbl, we extracted data from Amplitude (www.amplitude.com) and Tableau usage logs (www.tableau.com) into Microsoft Excel. We used Microsoft Excel for all analyses. See Table 2 for user and usage definitions.

**Table 1.** Implementation Phases and Resulting Hubbl Capabilities

Feature	Description
**Pre-go-live development**	
What’s new	Central location for any incoming messages to users.
Task list	User-specific work items assigned by and monitored by institutional leadership.
Training (integration)	Mobile integration with our learning management system for user-specific training schedule, including go-live related meetings assigned by institutional leadership.
Schedule	General go-live preparation and post-go-live meeting schedule.
Status	Countdown timer showing days, hours, and minutes until go-live.
Clinical corner	Central location for incoming messages targeting only clinicians. Messages can be saved for reference.
**Go-live development**	
Help desk ticket system (integration)	Mobile integration with our support and feature request ticket system, in addition to allowing ticket entry into central web-based support system.
Mission control	Support evaluation, user-specific list of groups, as well as link to the program website and FAQs (frequently asked questions).
**Post-go-live development**	
Tip sheets (integration)	Mobile integration with our content management system for any customer support documentation.

**Table 2.** User and usage definitions

Users and use	Definition
Enrolled users	Number of users who logged at least one event during the implementation.
Unique users	A distinct individual to whom events are attributed tracked by ID.
Active users	A user who has logged at least one event during per month.
Events	A user selection of a given capacity or feature.
Open rate	The number of communications opened as a percentage of total communications sent to each user.
Active use	Logging in and clicking within the app at least once.

### Results

**Figure 2.**
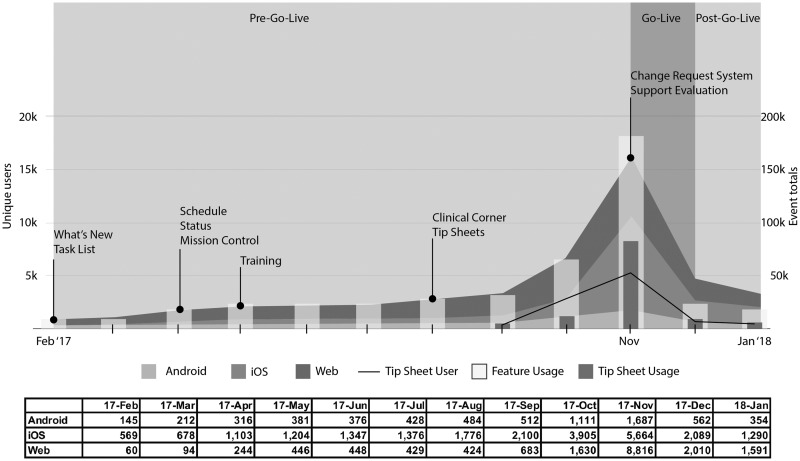
Hubbl active users and feature usage over time. Unique user is a distinct individual to whom events are attributed, tracked by device ID. Event totals are the total number of selections by unique users per each capability.

Figure 2 provides a summary of our experience with Hubbl from pre-go-live to January 31, 2018. The number of enrolled users steadily increased from February 2017 when Hubbl launched, through September 2017 1 month before go-live. An average of 1727 (median = 1515) users actively used Hubbl. Enrollment increased through the pre-go-live period, with an increase in both enrolled and active users just before go-live. Within a month before go-live from October 2017 to November 2017, the mobile and web users increase 9.52% (95% confidence interval CI [9.04%–10.0%]) and 29.3% (95% CI [28.7%–30.0%]), respectively, with a greater increase among web users versus smartphone users (*P* < 10e-6). The increasing trend continued for 2 weeks post-go-live (Android = +194, iOS = +647, web = +502). Within a month of go-live, usage decreased precipitously to 80% of the peak. In particular, from November 2017 to December 2017, the mobile users decrease 19.2% (95% CI [18.7%–19.7%]) and the web users decrease 27.8% (=6806/24518) (95% CI [27.1%–28.4%]). The web users’ rate on the month after go-live remained higher than the month before go-live (8.20% in December vs 6.6% in October, *P* < 10e-6), while mobile users rate dropped to lower rate (10.8% in December vs 20.5% in October, *P* < 10e-6).

The average pre-go-live session length was 2.5 minutes. Session length increased to 3 minutes and 47 seconds after go-live. The average session length was consistently highest for the web application and peaked at 9 minutes and 54 seconds 3 days after go-live.

### Pre-go-live usage

During the pre-go-live period, the *What’s new*, *Task list*, and *Training* components averaged 1053 user events/day. The number of total events during go-live and post-go-live increased to 1012, 721.

#### Communication

Over 97 communications were sent between February and October of 2017. These communications provided updates of serious issues or wins around the system. One example of communication sent out by VUMC leadership was a link to the implementation program terminology. Users opened an average of 19.5% of the communications in March and 7.7% in October. The number of messages created during go-live (November 2–November 30, 2017) was 39, and the average open rate was 3.66% (median = 2.43%).

#### Task lists

Over 727 tasks were created, assigned, and monitored for completion by operational leadership throughout the go-live phases. Before go-live there were 578 workflow tasks created and 67 nonworkflow tasks created. After go-live there were 55 nonworkflow tasks created. Our 485-site adult medical enterprise had a 96% task completion rate, while the 104-site pediatric medical enterprise had a 99% task completion rate. The patient access and revenue cycle enterprise consisting of 51 sites had an 89% task completion rate. Finally, the behavioral health enterprise consisting of 23 sites had a 98% task completion rate.

### Go-live

During go-live period, the top three features measured by total clicks into the capability, that were used were what’s new (48 865), Change request ticketing system—Pegasus (40 609), and post-implementation FAQs—tip sheets (34 728). Beyond the top three capabilities, the next highest capability was Schedule (5251).

Other functionality allowed the ability of just-in-time access to reference materials at a user’s fingertips. The reference materials provided by our training team served to assist users with troubling workflows or other operational issues during go-live. The searchable gateway allowed the user to enter a phrase and search the document repository. This repository was continuously updated to help guide end-users through the various functionality available with a new EHR. This dynamic repository offered a powerful opportunity for users to refer to support documents on both mobile and web clients; however, end-user feedback called to our attention that “Tip sheets are getting difficult to locate in Hubbl.” We enhanced our search and directory structure by allowing favorites and enhancing search across multiple parameters such as title, text, and metadata, and this is an opportunity for further improvement. The searchable gateway at its peak during the go-live period received over 80 000 searches, and it continues to be used with 16 992 monthly searches performed as recently as April 2019 during our latest upgrade.

#### End user change requests

During November, users created 5262 change request tickets via Hubbl. This was 12.8% of the overall number of tickets (41 017) created. The average time to resolve an issue was 6105 minutes or roughly 4.24 days for tickets created in Hubbl. Our ticket open rate continuously decreased from Go-Live, peaking at 7292 open tickets 1 week after go-live and decreasing to about 3000 a month after go-live.

#### Support evaluation

For 15 days after go-live, we asked a subset of our leadership team to complete a survey describing the satisfaction with our EHR each day. Questions included “How satisfied are EHR provider users (ie, MDs, NPs) at your site today?”, “How well is your site using the EHR today?”, and “How satisfied are the EHR non-provider users (ie, nurses, medical assistants) at your site today?” Responses ranged from 1 to 5, with 1 being very dissatisfied and 5 being very satisfied.

From November 2, 2017 through November 17, 2017, users on iOS filled out the EHR evaluation more times and by more users than Android or the web platforms. The web platform averaged 24% of the total usage. Leaders responded with an average initial rating of 1.8, 1.8, and 1.9, respectively, on a scale of 1–5, 5 being the best designation. The ratings increased to 3.5, 3.9, and 3.7, respectively, at the end of the survey. This data was found to be significant with a *P* < .001.

The FAQ and tip sheets functionality was released on August 31, 2017. Prior to go-live, 1003 tip sheets were created. In September 2017 tip sheets had 361 unique users who accessed the capability 1700 times and in October 2017 the capability had 3202 users who accessed the capability 18 571 times. Between November 2 and November 30, 2017, 428 additional Tip sheets were created in response to go-live issues or gaps in training. During this period, 5437 unique users accessed Tip sheets a total of 81 148 times. During the post-go-live period, 359 tip sheets were created. In December and January post-go-live, 941 and 670 unique users accessed the capability 6790 and 5218 times, respectively.

During this period, users across all platforms accessed this capability during this period and iOS users averaged 43.6% of the usage for this capability.

## DISCUSSION

Figure 2 shows that our system had relatively slow uptake of adopters until the final weeks before go-live, even though our communications team used multiple media to advertise Hubbl’s robustness and functionality. The slow adoption of the platform potentially led to a decrease in important operational communication or EHR functionality communication to stakeholders; however, this was combated with communication through e-mail, office staff, and notable fliers around campus.

Spreadsheets, documents, and e-mail correspondence are typical and essential communication modalities for dissemination of information throughout any implementation. The widespread adoption of Hubbl demonstrated that using an integrated tool for communication and task coordination allowed us to eliminate spreadsheets and other tracking documents, which are less secure and less adaptable data collection paradigms.

One unique capability Hubbl provided was the ability to receive rapid and ongoing daily feedback from frontline users at each site. Our daily survey results showed consistent provider and nonprovider satisfaction improvement with the newly implemented EHR, and leadership used this information to prioritize at the elbow (ATE) resource support. As an example, and due to our ability to collect feedback rapidly and regularly, if an ambulatory clinic site noted a substantial improvement in EHR usability and satisfaction, leadership would see the progression from clinic feedback and then could mobilize ATE resources to another location.

Assessing the return on the investment of any software is tricky, especially when the goal of the project is to consolidate existing tools. Our costs began with establishing a cross-functional governance structure consisting of operational, technology, and executive implementation leadership and committed one development team consisting of one product manager, five developers, and one quality analyst for an entire year at an initial estimated cost of $500 000 for the development team. From this effort, we achieved the described results and acquired four capabilities—a communication infrastructure, a distributed task management system, a content management user interface for EHR tip sheets, and a mobile help desk support ticket user interface. Our recurring costs of maintenance are estimated at less than $10 000 per year for maintenance releases. Users continue to utilize the content management and help desk support ticket systems today. Our training staff continues to maintain tip sheets through Hubbl instead of functionality available in our EHR because its ease of use. Since we completed the substantial development efforts in December 2017, we have had limited maintenance costs, other than an upgrade to our content management strategy. Users continue to rely on Hubbl both for data retrieval and for data aggregation. We believe benefits since before going live have more than justified the expenses to date, but we have not formally completed an ROI. Overall, our experiences with this application validated our belief in the value of integrating multiple communication modalities in one location to help with change management at the medical enterprise scale. We believe this experience is likely to generalize at other institutions of equal or lesser size.

## LIMITATIONS

We highlight several limitations in the creation, implementation, and usability of Hubbl. We used a rapid and iterative process to develop Hubbl so that the product aligned with our end-user communication and training implementation timeline. Thus, we were unable to capture a large amount of formal user feedback and evaluation and feedback of Hubbl. We created this tool specifically to assist with a medical enterprise-level implementation of an EHR and has not yet been used and tested with smaller implementations.

Due to the upgrade releases of Hubbl, users of the mobile application were required to re-enter their credentials after each upgrade. Individuals potentially did not receive push notifications because Hubbl may have logged them out at that time. The product team was developing new capabilities so quickly that we were unable to ensure backwards compatibility with previous app versions. This development restriction was felt to be a substantial barrier. Future product teams should consider and develop for backwards compatibility into their process.

Hubbl, due to development time constraints, could not offer direct access to the end-users required training courses in the mobile application. Instead, Hubbl provided a hyperlink to view the user’s required training. There was some feedback from early adopters that this feature was important for the mobile version, although the desktop version of the app offered direct access to the training web portal and the ability to complete the end user’s training. This limitation likely impacted usefulness of the app and would be an important addition to subsequent versions of Hubbl.

## CONCLUSION

In our experience with Hubbl, the use of a unified mobile and web-based communication platform during a substantial IT implementation is a feasible way to ensure task completion, project coordination, and timely information dissemination. While still in the early phases of distribution, this study suggests that tools such as Hubbl could be an essential component of just-in-time communication, task coordination, and change management.

## FUNDING

This research received no specific grant from any funding agency in the public, commercial, or not-for-profit sectors.

## AUTHOR CONTRIBUTIONS

All authors attested that they have made substantial contributions.
